# Nutritional habits, inhibitory control, and emotional reactivity to healthy and unhealthy food cues in non-obese female students: insights from heart rate variability

**DOI:** 10.3389/fnut.2025.1622087

**Published:** 2025-09-03

**Authors:** Michela Sarlo, Fiorella Del Popolo Cristaldi, Giulia Buodo, Carmen Belacchi

**Affiliations:** ^1^Department of Communication Sciences, Humanities and International Studies, University of Urbino Carlo Bo, Urbino, Italy; ^2^Department of General Psychology, University of Padova, Padova, Italy

**Keywords:** food cues, self-regulation, nutritional habits, heart rate variability, emotional reactivity, inhibitory control, Go/NoGo

## Abstract

**Background:**

Research shows that the nutritional habits of university students do not follow the national recommendations. While most studies have focused on the increased risk of overweight/obesity, avoiding unhealthy food or maintaining a normal weight does not necessarily result in a regular consumption of healthy essential nutrients.

**Methods:**

The present study was aimed at investigating the interplay between emotional reactivity and inhibitory control in 42 non-obese female students exposed to healthy (fish/lean meat, fruit/vegetables) and unhealthy (savory and sweet junk food) food pictures, after an average fasting of 7.5 h. Resting heart rate variability (HRV) was assessed as a physiological index of self-regulation, exploring its association with emotional reactivity and inhibitory control, as well as its predictive role in nutritional habits. We measured valence, arousal and craving during a free viewing time task and assessed inhibitory and attentional control through an emotional Go/NoGo task. Hunger, nutritional habits and frequency of physical activity were also collected.

**Results:**

Unhealthy foods elicited higher pleasantness, arousal and craving than healthy foods, indicating stronger appetitive motivation. Emotional reactivity was predicted by hunger or fasting duration as a function of food type. Higher HRV predicted slower reaction times to Go stimuli for all food types except fruit/vegetables. HRV and physical activity negatively predicted the habitual consumption of sweet junk food and positively predicted that of fruit/vegetables.

**Conclusion:**

Our results provide novel insights into the mechanisms underlying dietary self-regulation in non-obese female students, highlighting the significant role of resting HRV and physical activity in promoting healthy dietary choices and limiting junk food intake.

## 1 Introduction

Across Western countries, university students are often prone to poor dietary habits, including frequent consumption of ultra-processed, high-calorie foods, irregular meal patterns, and low daily intake of fruits and vegetables (1–4). Factors contributing to these behaviors include time constraints, stress, social influences, budget limitations, and the high availability of cheap fast food options in on-campus vending machines (5–8). Importantly, these eating habits often persist throughout the entire duration of university studies, posing the risk of extending into later life (6, 9).

While most research on this population has focused on the increased risk of overweight and obesity [see (10)], it is also recognized that, regardless of weight status, unbalanced diets with deficits in vitamin, mineral, and fiber intake play a significant role in promoting health issues (11–13). Furthermore, a lower-than-recommended intake of animal-protein sources, such as lean meat and fish, has been found to result in a reduced intake of vitamin B_12_, iron, zinc, and omega-3 fatty acids, while being associated with an increased consumption of foods high in sugar and fat (14). Overall, results across studies consistently indicate that most university students fail to meet the national dietary recommendations for food groups, e.g., (3, 4, 15, 16). A research by the Italian National Institute of Health (17, 18), investigating nutritional habits in a large sample of university students (*N* = 8,516), found that less than 45% consumed at least one portion of fruit per day and fewer than 23% ate at least two portions of vegetables per day. In addition, 59% ate fast foods only 1–2 times a month. Importantly, only 1.4% were obese, 9.8% were overweight, while 13.7% were underweight, which increased to 19.4% among female students, suggesting inadequate nutritional status.

To date, the psychological and neurobiological mechanisms hypothesized to underlie food choice and regulation of food intake are primarily based on research focused on overeating and obesity, e.g., (19–22). The interplay between bottom-up reward responsiveness, including automatic affective reactions and attentional bias to food cues, and top-down inhibitory control appears to be a key factor in the self-regulation of eating behavior (23–26). In particular, neuroimaging studies have shown that overconsumption of palatable, high-calorie foods is associated with increased responsivity of brain reward and motivation circuits (e.g., nucleus accumbens, orbitofrontal cortex, amygdala), and/or with reduced activation of inhibitory control regions (e.g., dorsolateral prefrontal cortex) [see (21, 27, 28), for a review].

However, avoiding unhealthy foods or maintaining a normal weight does not necessarily result in a regular consumption of healthy foods and essential nutrients. Indeed, the cognitive and emotional processes underlying the choice and consumption of healthy foods remain underexplored, and the neural correlates of low-calorie food processing have received little attention in the literature [see (29)]. Unlike palatable, high-calorie food, healthy food does not typically trigger intense craving or provide immediate hedonic rewards (30–32). Recent research (33, 34) suggests that motivational processes driving healthy food choices are strongly related to anticipated positive emotions and long-term outcomes, rather than to immediate emotions related to eating. Regarding the involved cognitive processes, inhibitory control seems to play a limited role in healthy food consumption, with studies failing to find consistent associations with healthy eating behaviors, such as consumption of fruit and vegetables (35–37), or non-fatty foods (38). Therefore, while inhibitory control plays a significant role in restraining prepotent responses to high reward foods (37, 39–41), healthy food choices may require the recruitment of executive processes that actively promote the desired response, such as action planning (42), or updating and monitoring goals (37).

Research in the last two decades has increasingly recognized heart rate variability (HRV) as a reliable physiological index of top-down self-control or self-regulation, including both emotional and behavioral components [(43), for reviews see (44, 45)]. The process of emotional regulation is aimed at modulating the intensity, type, and timing of emotional responses, with changes at self-reported, behavioral, and/or physiological levels (46, 47). Behavioral regulation, on the other hand, is aimed at achieving and maintaining specific goals through executive processes, including working memory, inhibitory control, and attentional control (45, 48). HRV, corresponding to the variation in the time intervals between consecutive heartbeats, reflects the autonomic regulation of the cardiac sinoatrial node, which at rest is predominantly influenced by parasympathetic (vagal) control (49). In the light of the Neurovisceral Integration Model (43), vagally-mediated resting HRV reflects the inhibitory influence of the prefrontal cortex on subcortical brain structures, which flexibly regulates cognitive, emotional, and behavioral responses to support goal-directed behavior. Moreover, converging evidence from experimental and clinical research highlights the close interplay between vagal activity and interoceptive processes, suggesting that emotional and behavioral regulation is grounded in the integrated perception of internal bodily signals. This perspective aligns with current views of HRV as not only an index of prefrontal inhibitory control but also as a physiological correlate of interoceptive function, supporting flexible and adaptive responses to environmental demands [see (50), for a review].

On these grounds, lower resting HRV was reported in individuals with obesity and binge eating (51, 52), and was related to greater food craving and overeating (53, 54). Conversely, higher HRV was associated with greater self-control in a challenging food-choice task (55) and with successful weight loss by diet (51–56). However, the potential effects of differences in body mass index (BMI), dietary restrictions, fasting status, and lifestyle habits, such as physical activity, are often overlooked in the relevant literature [see (57)], particularly in studies involving non-clinical, normal-weight samples. Crucially, it remains unclear whether HRV is more strongly related to the emotional or behavioral components of top-down self-regulation, or both. This seems particularly relevant for unhealthy eating behaviors, where emotional responses, inhibitory control, and goal-directed behavior play a critical role in shaping long-term dietary choices.

The present study was aimed at investigating the interplay between emotional and behavioral regulation in predicting the habitual consumption of healthy and unhealthy foods in a sample of non-obese female students. Resting HRV was assessed as a physiological index of self-regulation, exploring its association with emotional reactivity and inhibitory control during the viewing of healthy and unhealthy food pictures, as well as its predictive role in nutritional habits. An emotional Go/NoGo task using food cues was employed to obtain measures of inhibitory control (i.e., commission errors on NoGo trials) and attentional task engagement (i.e., omission errors on Go trials), as well as indices of approach-related behavior and attentional control (i.e., reaction times on Go trials) [see (58)]. Ratings of valence, arousal, and craving were collected to assess emotional reactivity to food pictures during a free viewing time task, which additionally provides an overt index of attention (59). Nutritional habits were assessed to evaluate the habitual consumption of different healthy and unhealthy foods, and their potential associations with resting HRV and with emotional and behavioral regulation processes. Lastly, the contribution of BMI, physical activity, food deprivation duration, and perceived hunger was investigated to account for their potential effects on the relationships of interest.

We hypothesized that unhealthy food stimuli would result in higher ratings of pleasantness, arousal, and craving, as well as lower inhibitory control, as indexed by higher commission errors during the Go/NoGo task. In contrast, healthy foods were expected to elicit lower emotional reactivity and less task interference. Moreover, we anticipated that the habitual consumption of unhealthy foods would be positively associated with greater emotional reactivity and lower inhibitory control, whereas the consumption of healthy foods would be less influenced by immediate emotional or behavioral self-regulation, being more closely related to long-term motivations and outcomes. Finally, individual differences in HRV were expected to play a significant role in these processes. Specifically, higher resting HRV was hypothesized to be associated with lower emotional reactivity and better inhibitory control during exposure to unhealthy foods, as well as with lower habitual consumption of unhealthy foods. Higher HRV might also actively support healthy dietary habits by prioritizing higher order goals during the processing of food choices.

Overall, the present research aimed to provide novel insights into the emotional and behavioral self-regulatory mechanisms that underlie food-related decisions and shape habitual dietary patterns in non-obese female university students, a population often prone to suboptimal nutritional choices. By integrating psychophysiological and behavioral measures, our findings may contribute to a better understanding of self-regulation and help inform future research aimed at promoting healthier eating habits.

## 2 Materials and methods

### 2.1 Participants

Forty-four female students at the University of Urbino Carlo Bo were recruited through social network advertisements and campus flyers. Only women were recruited since research has shown that they are more responsive to visual food-related stimuli [e.g., (60, 61)].

Data from two participants were excluded from the final analyses because they were deemed outliers on HRV measurements (> 2.5 SDs from the mean, based on the root mean square of successive interval differences, RMSSD), leaving a final sample of 42 participants (mean age = 21.90 years, SD = 3.12, range = 18–34). Mean RMSSD for the final sample was 41.12 ms (SD = 18.23, range = 16.92–85.64). Mean BMI was 20.25 kg/m^2^ (SD = 2.30, range = 16.82–26.14); among participants, 28 (67%) were normal-weighted (BMI ≥ 18.5 and < 25 kg/m^2^), 12 (28%) were underweighted (BMI < 18.5 kg/m^2^), and 2 (5%) were overweighted (BMI ≥ 25 and < 30 kg/m^2^).

Participants were included if they had a BMI < 30 kg/m^2^ (i.e., not obese) and followed an omnivore diet. Exclusion criteria included adherence to any special diet, presence of alimentary disorders, allergies or intolerances, a history of cardiovascular, neurological, or psychiatric conditions, or use of medications influencing cardiovascular or central nervous system function. Medication use was assessed through general screening questions, and no participants reported current medication use. Hormonal contraceptive use was not specifically addressed and was not spontaneously reported by any participant.

### 2.2 Procedure

Participants were recruited through an online form including the study description, preliminary informed consent, inclusion/exclusion criteria, and an *ad hoc* questionnaire on nutritional habits and frequency of physical activity. Volunteers fulfilling the study criteria were contacted to schedule the experimental session. Participants were instructed to refrain from eating and from consuming any drink (except water) for at least 3 h before arriving at the appointment. This limit was selected based on previous studies [e.g., (60, 61)]. To check for compliance and to record the duration of food deprivation, participants were asked to indicate the exact time at which they had finished their last meal.

Upon arrival, each participant read and signed an informed consent form and was then seated on a comfortable chair in a dimly lit, sound-attenuated room. After a 10-min adaptation period, the E4 device (Empatica, Milan, Italy) was placed on the participant’s left wrist, following the manufacturer’s instructions, and physiological signals were recorded in streaming mode (via Bluetooth) for 5 min. During measurement, participants sat still, with eyes open. Before starting the experimental tasks, participants were asked to rate how hungry they felt on a 1–9 scale (1 = not hungry at all, 9 = extremely hungry). Then, they performed the Go/NoGo task, followed by the free viewing time/emotional rating task. At the end of the experimental session, participants were thanked and debriefed. To summarize, the temporal structure of data collection was as follows:

- Nutritional habits and frequency of physical activity were assessed via an online questionnaire prior to the experimental session.
- Resting HRV was recorded for 5 min at the start of the session.
- Hunger ratings were collected immediately before the Go/NoGo task.
- Behavioral Go/NoGo data and free viewing times, along with subjective emotional ratings, were collected sequentially during their respective tasks.

### 2.3 Food stimuli

The employed stimuli consisted of 120 food pictures depicting healthy (*n* = 60) or unhealthy (*n* = 60) foods. Healthy foods are defined as those rich in minerals, fibers, vitamins, high-quality proteins, and unsaturated fats, while containing low levels of saturated fats and sodium. Unhealthy foods, often referred to as junk foods, are defined as ultra-processed, with low nutritional value (i.e., lacking in vitamins, minerals, and fibers), and high in sugars, saturated fats, artificial additives and preservatives. Based on these definitions [e.g., (62, 63)], healthy foods included *fruits/vegetables* (fruit salads or skewers, raw vegetable salads, cooked-vegetable dishes; *n* = 30) and *fish/lean meat* (different cuts of fresh fish or lean red/white meat prepared in different cooking styles; *n* = 30). Unhealthy foods included *savory junk food* (pizza, cheeseburger, French fries, salty snacks; *n* = 30), and *sweet junk food* (donuts, chocolate cookies, prepackaged ice-cream and sweet snacks; *n* = 30).

All individual pictures were selected from the web and show close-up views of food items. Some of them were employed in previous studies by our group (64, 65). They were sourced from publicly available internet resources over several years and used for experimental purposes under fair use considerations. Copyright restrictions and lack of detailed license information prevent sharing the full set as Supplementary material. However, images are available upon reasonable request, with the user responsible for complying with copyright laws.

### 2.4 Emotional Go/NoGo task

Each picture was surrounded by a colored frame (pink or blue) that cued the participant to either press a key (Go trials) or withhold the response (NoGo trials). Frame colors indicating Go and NoGo trials were counterbalanced across participants. For each food category, the percentage of Go and NoGo cues was 70 and 30%, respectively. Each picture was presented three times, for a total of 360 trials (252 Go and 108 NoGo). The stimuli were presented in semi-random order (i.e., no consecutive NoGo trials) in two blocks of 180 trials each. Each trial began with a 500-ms white central fixation cross on a black background, followed by the presentation of the framed picture for 600 ms. The inter-trial interval varied randomly between 500 and 800 ms.

Participants were instructed to press a key with their index finger as rapidly and accurately as possible whenever a picture with the Go color frame was presented, and to withhold pressing the key when the picture had a NoGo color frame. They were asked to maintain fixation on the center of the screen throughout the task and were allowed to rest between the two experimental blocks. Eight practice trials, with pictures depicting foods unrelated to the selected experimental categories (e.g., pasta, cheese), were presented before the beginning of the experimental session. The task was presented on a 19-inch computer screen through a PC running E-prime 3.0 software (Psychology Software Tools, Pittsburgh, PA, United States), at a viewing distance of 1 m.

### 2.5 Free viewing time task and emotional ratings

Participants were presented with a subset of pictures (6 pictures for each food subtype, for a total of 24 pictures) with no colored frame. They were allowed to watch each picture as long as they wanted, being instructed to press a key to stop picture presentation. After the offset of each picture, participants were required to rate the emotional state experienced during picture viewing on the 1–9 point scales of Valence (unpleasantness/pleasantness) and Arousal (calm/activation), using a computerized version of the Self-Assessment Manikin [SAM; (66)]. They were also asked to rate their desire to eat each specific food displayed, using a computerized version of the 1–9 point scale of the SAM food craving (67), ranging from a face with a mouth shut to a face with a drooling mouth. For each SAM scale, 9 represents a high rating (i.e., high pleasure, high arousal, high craving), and 1 represents a low rating (i.e., low pleasure, low arousal, low craving).

### 2.6 *Ad hoc* questionnaire on nutritional habits

In order to get a measure of participants’ nutritional habits, we developed an *ad hoc* questionnaire drawn by that validated by the Italian National Institute of Health (17). The questionnaire included queries about the habitual consumption of fruit, vegetables, fish, lean meat, savory and sweet junk food using a 0–6 scale (0 = never, 1 = rarely, 2 = once per week, 3 = two-four days per week, 4 = five-six days per week, 5 = once per day, 6 = more than once per day). Other items referred to demographics, weight and height, and frequency of physical activity (0 = never, 1 = once per month, 2 = once per week, 3 = two-three days per week, 4 = four-six days per week, 5 = everyday).

### 2.7 Physiological recording and HRV computation

Inter-beat Interval (IBI) time series were derived from the blood volume pulse (BVP) signal recorded by the E4 wrist-worn device (sampling frequency: 64 Hz, resolution: 0.9 nW/digit). BVP raw data were exported using the Empatica Connect web application. IBIs were estimated from the pulse intervals (i.e., the distances between pulse wave foot points) of the BVP signal. The *findpeaks* function of the “pracma” R pack-age (68) was used to automatically detect the IBI time series [see (69)]. The IBI series were preprocessed for artifact removal using automatic procedures, followed by interactive visual inspection, as recommended (70). Artifact correction and interpolation, and HRV analyses were performed using the Kubios HRV Scientific 4.1.0 software (Oy, Kuopio, Finland). In the time domain, the RMSSD (in ms) was computed as a measure of HRV.

### 2.8 Statistical analysis

Separate repeated-measures analyses of variance (ANOVAs) were conducted on mean omission and commission error rates, reaction times (RTs) to Go trials, ratings of valence, arousal and craving, and viewing times, with *Food Type* (fruit/vegetables, fish/lean meat, savory junk food, and sweet junk food) as within-subjects factor. To control for type I error, the Greenhouse–Geisser (G-G) correction was applied when necessary. In the text, the uncorrected degrees of freedom are reported together with the adjusted probability values. Tukey HSD post-hoc tests were employed to further examine significant effects (*p* < 0.05). Bayesian analyses were conducted for Go/NoGo performance indices, where they provide additional insight into the strength of evidence for or against subtle condition effects.

Pearson’s correlations were used to explore the association between variables. Hierarchical regression analyses were conducted separately for each food type to test the influence of resting HRV (RMSSD) on emotional reactivity and performance in the Go/NoGo task. To reduce the number of variables, and given that ratings of valence, arousal, and craving were highly correlated (see Supplementary Table S1) and showed no differential effects on the ANOVAs (see Results), a composite index of emotional reactivity was derived by summing the ratings across these dimensions. Given the differences in predictors across analyses, the models were specified as follows:

- For emotional reactivity as the dependent variable, model 1 included BMI, deprivation duration, and hunger; model 2 (final model) added HRV.
- For Go RTs and commission error rates as dependent variables, model 1 included BMI, deprivation duration, and hunger; model 2 added emotional reactivity; model 3 (final model) added HRV.

To assess the predictors of habitual consumption of healthy and unhealthy foods, separate hierarchical regression analyses were conducted for each food type:

- For healthy foods (fruit/vegetables and fish/lean meat), model 1 included BMI and physical activity; model 2 added emotional reactivity (composite index for each food type) and inhibitory control over unhealthy food (mean commission errors across savory and sweet junk food); model 3 (final model) added HRV.
- For unhealthy foods (savory and sweet junk food), model 1 included BMI and physical activity; model 2 added emotional reactivity and inhibitory control specific to the corresponding junk food type; model 3 (final model) added HRV.

In all regression analyses, HRV was entered in the final Model to assess its incremental contribution after controlling for other relevant predictors, in line with the main hypotheses of the study. Covariates were selected based on theoretical relevance and consistency with prior research.

All statistical analyses were performed using IBM SPSS Statistics (version 29.0), except for post-hoc Tukey HSD tests following ANOVAs, which were carried out using TIBCO® Statistica (version 14.0).

Sensitivity analyses conducted with G*Power 3.1 indicated that, with the current sample size (*N* = 42), the repeated-measures ANOVA had 80% power to detect medium-sized effects (*f* = 0.18, *α* = 0.05). For the multiple regression models, the minimal detectable effect size was *f*^2^ = 0.19 for a single predictor (HRV) added incrementally to a model including the covariates described above. These values suggest that the study was sufficiently powered to detect moderate effects, while smaller effects may have gone undetected.

## 3 Results

### 3.1 Food deprivation duration and hunger ratings

Participants reported a mean food deprivation duration of 7.46 h (SD = 4.60, range = 3–15) and a mean hunger rating of 5.71 on a 1–9 scale (SD = 1.64, range 1–8). No significant correlation was found between these two variables (*p* > 0.79; see Supplementary Table S1).

### 3.2 Performance on the emotional Go/NoGo task

The ANOVA on mean Go RTs revealed a significant main effect of *Food Type* [*F* (3,123) = 5.16, *p* = 0.007, *ε* = 0.68, *η*^2^_p_ = 0.11]. Bayesian analysis yielded moderate evidence in favor of the alternative hypothesis (BF_10_ = 5.82). Post-hoc tests showed that RTs were significantly slower for savory junk food and fruit/vegetables compared to fish/lean meat (*p* < 0.03 and *p* < 0.002, respectively). No significant differences emerged among the other food types.

No significant effects were obtained for commission error rates [*F* (3,123) = 1.54, *p* = 0.21, *ε* = 0.93, *η*^2^_p_ = 0.04] and omission error rates [*F* (3,123) = 0.19, *p* = 0.87, *ε* = 0.83, *η*^2^_p_ = 0.005]. Bayesian analyses strongly supported the null hypothesis for both measures, with Bayes factors (BF_10_) of 0.025 and 0.003, respectively, indicating strong to extreme evidence for the absence of food-type effects.

Means and SDs for all the behavioral measures are provided in Table 1.

**Table 1:** Means (and SDs) for the different measures of Go/NoGo task performance as a function of food type.

Food type	Go RTs (ms)	Omission errors (%)	Commission errors (%)
Fruit/vegetables	342 (36)	0.74 (1.58)	14.93 (11.53)
Fish/lean meat	333 (37)	0.62 (1.27)	12.26 (10.77)
Savory junk food	341 (35)	0.62 (1.32)	13.19 (10.34)
Sweet junk food	337 (38)	0.69 (1.47)	14.48 (11.14)

### 3.3 Emotional reactivity and viewing times

**Figure 1:**
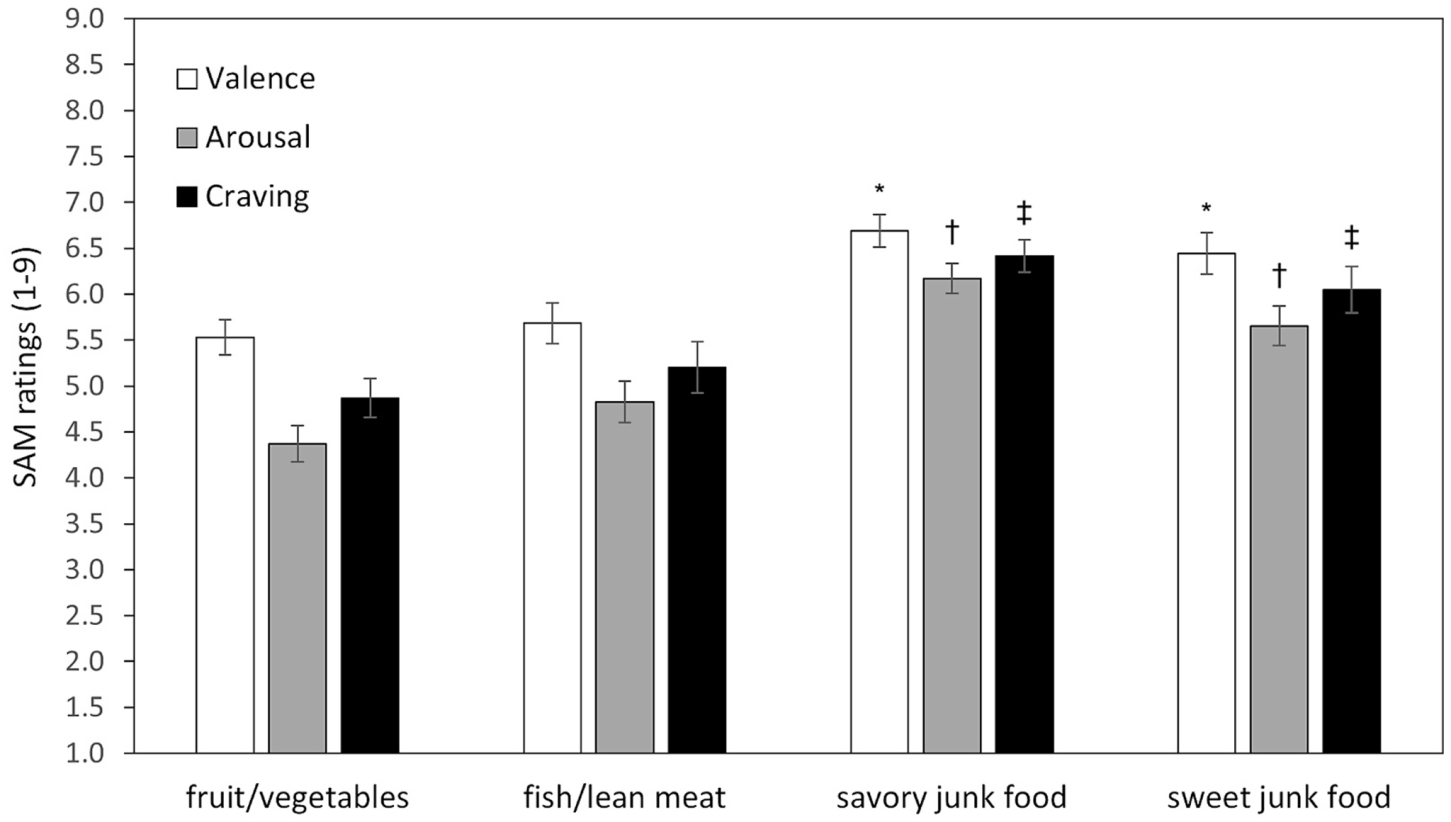
Mean ratings of valence, arousal, and craving by food type. Error bars represent standard errors of the means. Symbols above bars representing savory and sweet junk foods indicate statistically significant differences (all ps < 0.035) compared to each healthy food type (fruit/vegetables and fish/lean meat). Specifically, an asterisk (*) denotes significant differences for Valence, a dagger (†) for Arousal, and a double dagger (‡) for Craving. No significant differences were found between the two healthy food categories or between the two unhealthy food categories.

The ANOVA on emotional ratings revealed a significant main effect of *Food Type* for valence [*F* (3,123) = 8.13, *p* < 0.0001, *ε* = 0.74, *η*^2^_p_ = 0.17], arousal [*F* (3,123) = 18.04, *p* < 0.0001, *ε* = 0.81, *η*^2^_p_ = 0.31], and craving [*F* (3,123) = 11.02, *p* < 0.0001, *ε* = 0.73, *η*^2^_p_ = 0.21]. Post-hoc tests indicated that unhealthy foods (i.e., savory and sweet junk foods) elicited significantly higher pleasantness, arousal, and craving compared to healthy foods (i.e., fruit/vegetables and fish/lean meat) (all ps < 0.035). No significant differences were found between savory and sweet junk foods, or between fruit/vegetables and fish/lean meat (all ps > 0.22; Figure 1).

For free viewing times, no significant effects were found (*p* = 0.44). On average, food pictures were viewed for 7,460 ms.

### 3.4 Nutritional habits

**Figure 2:**
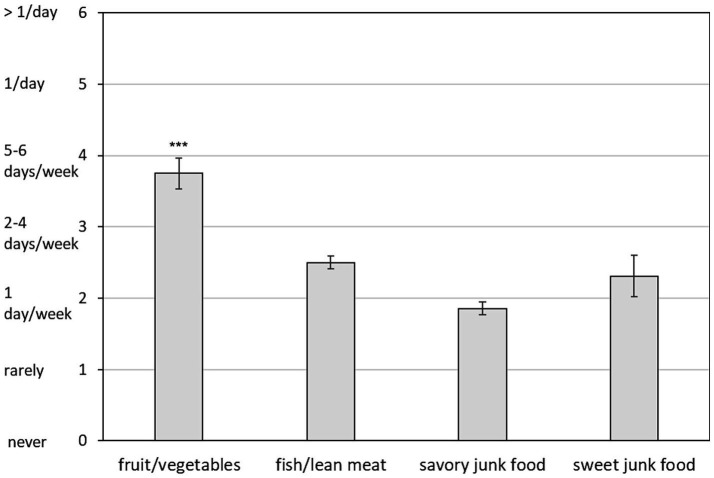
Mean self-reported frequency of consumption of healthy and unhealthy foods (0–6 scale: 0 = never, 1 = rarely, 2 = once per week, 3 = two-four days per week, 4 = five-six days per week, 5 = once per day, 6 = more than once per day). Error bars represent standard errors of the means. Asterisks (***) above the fruit/vegetables bar indicate significantly higher consumption compared to all other food types (*p* < 0.0001). No significant differences were found among fish/lean meat, savory junk food, and sweet junk food categories.

The ANOVA revealed a significant main effect of *Food Type* [*F* (3, 123) = 16.46, *p* < 0.0001, *ε* = 0.50, *η*^2^_p_ = 0.29]. Post-hoc comparisons indicated that the average self-reported consumption of fruits and vegetables was significantly higher than that of any other food type (all ps < 0.0001), with participants consuming these foods several days per week on average (Figure 2). While the reported consumption of savory and sweet junk foods was relatively low, equating to approximately once per week on average, no significant difference was found between the consumption of junk foods and fish/lean meat (all ps > 0.10).

### 3.5 Hierarchical regressions

To provide a comprehensive overview of the interrelationships among all variables under study, the full correlation matrix is available in the Supplementary Table S1. Variance inflation factor (VIF) checks for the hierarchical regression analyses showed values < 1.55, with tolerance levels > 0.64 across all models, indicating no evidence of multicollinearity.

#### 3.5.1 Emotional reactivity

For fruit/vegetables no significant regression models were found (all ps > 0.35). For fish/lean meat, only model 1 reached significance [*F* (3, 41) = 3.01, *p* = 0.042, *R*^2^ = 0.19], indicating that food deprivation negatively predicted emotional reactivity [*β* = −0.37, *t* (38) = −2.48, *p* = 0.018], while BMI and hunger were not retained in the model. The addition of HRV as an independent variable in model 2 did not yield a significant improvement (*p* = 0.082). Full regression output is reported in the Supplementary Table S2.

For savory junk food, only model 1 reached significance [*F* (3, 41) = 2.87, *p* = 0.049, *R*^2^ = 0.19], showing that hunger positively predicted emotional reactivity [*β* = 0.33, *t* (38) = 2.23, *p* = 0.032], while BMI and deprivation duration were not retained in the model. The inclusion of HRV in model 2 did not result in a significant improvement (*p* = 0.10). Full regression output is reported in Supplementary Table S3.

For sweet junk food, no significant regression models were obtained (all ps > 0.15).

#### 3.5.2 Reaction times to Go stimuli

For fruit/vegetables, no significant regression models were obtained (all ps > 0.14).

For fish/lean meat, only the final model was significant (F (5, 41) = 2.93, *p* = 0.026, *R*^2^ = 0.29), indicating that deprivation duration [*β* = 0.35, *t* (36) = 2.27, *p* = 0.029] and HRV [*β* = 0.43, *t* (36) = 2.95, *p* = 0.006] positively predicted Go RTs. The effect of BMI as a negative predictor approached significance (*p* = 0.056), while hunger and emotional reactivity were not retained as significant predictors. Full regression output is reported in Supplementary Table S4.

For savory junk food, only the final model was significant [*F* (5, 41) = 2.80, *p* = 0.031, *R*^2^ = 0.28], indicating that HRV was a positive predictor of Go RTs [*β* = 0.38, *t* (36) = 2.53, *p* = 0.016]. The effect of emotional reactivity as a positive predictor approached significance (*p* = 0.053), while BMI, deprivation duration, and hunger were not retained as significant predictors. Full regression output is reported in Supplementary Table S5.

For sweet junk food, only the final model was significant [*F* (5, 41) = 2.82, *p* = 0.030, *R*^2^ = 0.28], indicating that HRV positively predicted Go RTs [*β* = 0.37, *t* (36) = 2.49, *p* = 0.017]. The effect of deprivation duration as a positive predictor approached significance (*p* = 0.061), while BMI, hunger, and emotional reactivity were not significant predictors. Full regression output is reported in Supplementary Table S6.

#### 3.5.3 Commission errors

Regression analyses for commission errors did not yield any significant models (all ps > 0.36).

#### 3.5.4 Nutritional habits

**Figure 3:**
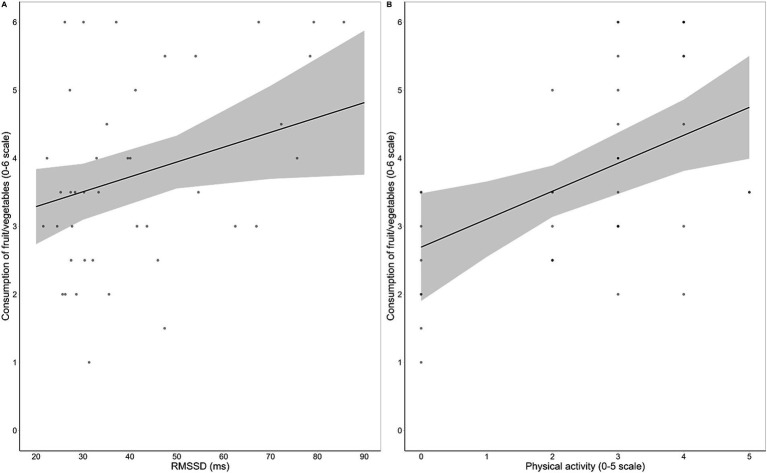
Regression scatterplots illustrating the significant positive associations between resting HRV, as measured by the root mean square of successive interval differences (RMSSD), and habitual consumption of fruit/vegetables **(A)**, and between frequency of physical activity and habitual consumption of fruit/vegetables **(B)**, with the regression lines and the standard errors of the fits superimposed.

For fruit/vegetables all three models were significant. The final model [*F* (5, 41) = 6.12, *p* < 0.001, *R*^2^ = 0.46] indicated that physical activity [*β* = 0.43, *t* (36) = 3.00, *p* = 0.005] and HRV [*β* = 0.29, *t* (36) = 2.16, *p* = 0.037] were positive predictors of habitual consumption (Figure 3), while BMI, emotional reactivity to fruit/vegetables, and inhibitory control over unhealthy food were not retained as significant predictors. Full regression output is reported in Supplementary Table S7.

For fish/lean meat, all three models were significant. The final model [*F* (5, 41) = 5.12, *p* = 0.001, *R*^2^ = 0.42] showed that physical activity [*β* = 0.36, *t* (36) = 2.49, *p* = 0.018] and emotional reactivity to fish/lean meat [*β* = 0.40, *t* (36) = 3.08, *p* = 0.004] were positive predictors of habitual consumption, while BMI, inhibitory control over unhealthy food, and HRV were not retained as significant predictors. Full regression output is reported in Supplementary Table S8.

**Figure 4:**
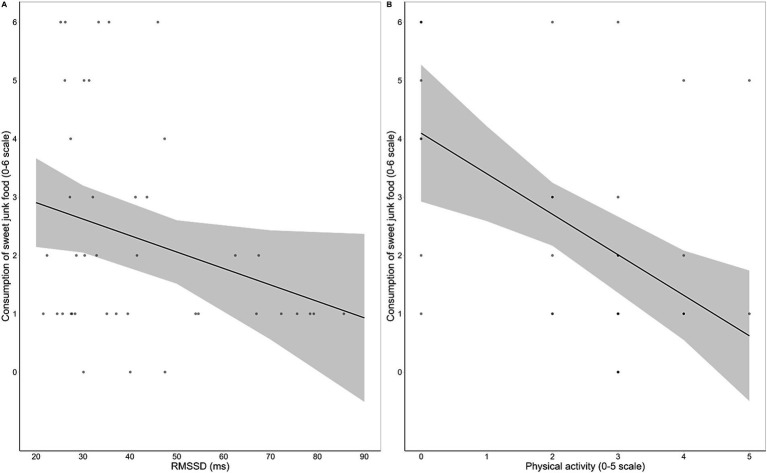
Regression scatterplots illustrating the significant negative associations between resting HRV, as measured by the root mean square of successive interval differences (RMSSD), and habitual consumption of sweet junk food **(A)**, and between frequency of physical activity and habitual consumption of sweet junk food **(B)**, with the regression lines and the standard errors of the fits superimposed.

Regarding the habitual consumption of unhealthy food, none of the regression models for savory junk food was significant (all ps > 0.55). In contrast, all three models for sweet junk food were significant. The final model [*F* (5, 41) = 4.90, *p* = 0.002, *R*^2^ = 0.41] indicated that physical activity [*β* = −0.54, *t* (36) = −3.39, *p* = 0.002] and HRV [*β* = −0.27, *t* (36) = −2.07, *p* = 0.046] were negative predictors of sweet junk food consumption (Figure 4). The remaining variables (BMI, emotional reactivity and inhibitory control over sweet junk food) did not significantly contribute to any model. Full regression output is reported in Supplementary Table S9.

## 4 Discussion

The nutritional habits reported by participants appear to be consistent with their BMI distribution, indicating a prevalence of normal-weight (67%) and a substantial proportion of underweight women (28%). Although fruits and vegetables were the most frequently reported food group, their consumption (almost 5–6 days per week, on average) falls well below the five portions per day recommended by the national dietary guidelines (71) and may contribute to insufficient intake of essential nutrients. Similarly, the reported consumption of fish and lean meat (approximately once per week) falls below the 2–3 times and 1–3 times, respectively, recommended by the national dietary guidelines (71), potentially leading to inadequate intake of high-quality proteins. Moreover, the average consumption of junk foods, while relatively low (approximately once per week), was comparable to the intake of fish/lean meat, suggesting that healthy protein sources were not prioritized, thus contributing to potential imbalance in participants’ diets. These results are consistent with the available literature in demonstrating that university students fail to meet the dietary recommendations for food groups (3, 4, 15, 16), while raising concerns about undernutrition, given the relatively high proportion of underweight women found in this and other Italian samples [see (17, 18)].

As expected, savory and sweet junk foods consistently elicited greater self-reported emotional reactivity (i.e., pleasantness, arousal, and craving) than healthy foods (fruit/vegetables and fish/lean meat), irrespective of the specific food type (Figure 2). This result is consistent with previous evidence [e.g., (25)] indicating that highly palatable, calorie-dense foods have higher hedonic and motivational appeal, due to their association with immediate energy gain and high reward value. However, emotional reactivity was unrelated either to attentional engagement, as measured by spontaneous viewing times and by RTs on Go trials, or to inhibitory control, as measured by commission errors on NoGo trials. Thus, the greater emotional reactivity found for unhealthy foods did not translate into longer viewing times or RTs overall. However, a near-significant (*p* = 0.053) predictive effect of emotional reactivity to savory junk food on RT slowing was observed, suggesting attentional interference exerted by emotional salience during response execution. The lack of significant differences in free viewing times among food types, together with the prolonged average viewing duration (> 7 s), suggests that, under food deprivation, food-related stimuli are intrinsically attention-grabbing, regardless of their emotional salience.

Our results also failed to demonstrate significant associations between self-reported emotional reactivity and inhibitory control. Specifically, commission errors did not differ across food types, and emotional reactivity did not emerge as a significant predictor in the regression models, suggesting that the heightened emotional reactivity elicited by junk foods did not impair the ability to inhibit prepotent responses. The average percentage of commission errors, ranging between 12.26 and 14.93%, suggests that the emotional Go/NoGo task was sufficiently challenging, but not hard. Additionally, the floor effect of the omission errors, averaging below 1% (see Table 1), indicates that participants maintained a high level of attention and promptness throughout the task, resulting in consistent performance across food types. Therefore, unlike previous studies by our group using the same Go/NoGo task with high-arousal pleasant and unpleasant pictures (72, 73), the emotional characteristics of food stimuli did not effectively modulate the top-down inhibitory mechanisms, even under moderate fasting.

On the other hand, self-reported emotional reactivity was influenced by current nutritional status. Specifically, food deprivation duration negatively predicted emotional reactivity to fish/lean meat (Supplementary Table S2), possibly reflecting less attraction to healthy protein-based foods, which offer less immediate energy, during fasting. Indeed, high-protein foods provide delayed satiety effects rather than immediate gratification (74). This interpretation is consistent with the finding that longer deprivation durations predicted slower Go RTs for this food type (Supplementary Table S4), suggesting that lower motivational priority reduced readiness for approach-related behavior. In contrast, hunger positively predicted emotional reactivity to savory junk food. This finding underscores the role of hunger in amplifying emotional salience of calorie-dense, highly palatable foods (75, 76), with savory items providing more immediate reward value than sweet ones [see (64)]. As a side remark, our data support the distinction between food deprivation, an objective indicator of energy balance, and self-reported hunger, a multifaceted construct shaped by visceral sensations, cognitive expectations, emotional states, and anticipatory reward processes (77). The lack of a significant correlation observed in the present study (see Supplementary Table S1), together with the weak to moderate correlations reported in the literature (78), suggests evidence for distinct underlying mechanisms, while highlighting the complexity of hunger as a multidimensional subjective experience.

The most relevant findings of this study concern the role of resting HRV in predicting nutritional habits and regulating attentional control to food stimuli. However, its influence was more complex than expected. In contrast with our hypothesis, higher HRV was not significantly associated with lower emotional reactivity or greater inhibitory control during exposure to unhealthy foods. Instead, its effects were observed on Go RTs during the Go/NoGo task, with higher HRV predicting slower responses for all food types except fruit/vegetables. This may reflect the engagement of “vagal brake” (79) in facilitating attentional control over automatic approach tendencies toward food cues, suggesting greater attentional self-regulation of motivational drive, rather than direct modulation of emotional responses or inhibitory control. This regulatory process seems to be unnecessary for fruit and vegetables, at least under moderate fasting. Interestingly, in the case of savory junk food, emotional reactivity also showed a marginal effect as a positive predictor, with emotional interference likely playing an additional role on RT slowing.

Crucially, our results provide significant evidence on the predictive role of resting HRV in regulating dietary behaviors. Independent of BMI, physical activity, emotional reactivity to food cues, and inhibitory control ability over unhealthy food, higher HRV was associated with healthier nutritional habits, including increased consumption of fruits and vegetables (Figure 3A) and reduced intake of sweet junk food (Figure 4A). These results seem inconsistent with the effects of HRV observed on Go RTs, suggesting that distinct mechanisms underlie its influence on immediate responses to food cues and long-term nutritional habits. HRV might generally promote immediate attentional control and motivational regulation during the processing of food stimuli, while also supporting long-term self-regulation in maintaining healthy dietary goals and resisting dietary temptation. However, habitual food consumption is influenced by multiple mechanisms, with factors such as lifestyle habits, convenience, time, cost, and food accessibility likely playing a critical role beyond physiological self-regulation. This might be the case for fish and meat, which are typically more expensive and less accessible to students than fruits, vegetables, or processed foods. Similarly, savory junk food, as compared to sweets, often represents a more suitable meal-like option when eating outside home. Therefore, the long-term regulatory influence of HRV may be limited for foods whose consumption is more driven by external factors.

Our data also underscore the significant impact of physical activity on the habitual consumption of both healthy and unhealthy foods. Specifically, the frequency of physical activity consistently emerged as a strong positive predictor of fruit/vegetables (Figure 3B) and fish/lean meat consumption, and a negative predictor of sweet junk food intake (Figure 4B). Physical activity is known to contribute to the prevention of weight gain through increased energy expenditure (80) and, notably, through appetite regulation in normal-weight individuals (81). In particular, regular exercise has been associated with preference for low-fat foods (82) and decreased neuronal responses to food cues with high hedonic value (83). Our findings nicely fit with this evidence by demonstrating that physical activity not only is associated with a limitation of sweet junk food intake, but also actively promotes the consumption of different kinds of healthy food.

### 4.1 Limitations and future directions

Some limitations of this study should be acknowledged. First, the sample was limited to female participants. While this choice was based on their greater responsiveness to visual food cues (84, 85) and higher underweight risk compared to men (17, 18), the gender specificity of the sample represents a limitation for the generalizability of the findings to mixed or male populations. Additionally, hormonal contraceptive use was not specifically assessed and may have modest effects on cardiovascular and emotional regulation, potentially influencing study outcomes. Second, the Go/NoGo task employed may lack sensitivity in effectively capturing the interplay between inhibitory performance and emotional reactivity, which was otherwise assessed only through explicit, self-report measures. Lastly, while HRV was used as a predictor of self-regulation and nutritional habits, it is also influenced by lifestyle factors such as sleep quality, stress levels, and health status (70). Although physical activity was accounted for in our analyses, the bidirectional relationship between HRV and lifestyle factors makes it difficult to determine causality.

Future research should explore potential sex-related differences in the self-regulatory mechanisms underlying eating behaviors, while using more cognitively demanding tasks and including multidimensional measures of emotional reactivity. In this context, alternative paradigms with greater parametric sensitivity to inhibitory processes (e.g., an emotional stop-signal task) could be employed, or neural measures such as event-related potentials could be integrated to gain additional information on the temporal dynamics of response inhibition. Moreover, longitudinal or intervention-based designs could help clarify the causal direction of the relationship between HRV, lifestyle factors, and nutritional habits.

## 5 Conclusion

The present study provides novel insights into the mechanisms underlying food self-regulation in non-obese, predominantly normal-weight university students, highlighting the significant role of resting HRV and physical activity in promoting healthy dietary choices and limiting junk food intake. Distinct effects of HRV were found on nutritional habits and attentional control during exposure to food cues, suggesting a complex interplay between long-term and immediate regulatory processes. However, HRV did not predict inhibitory control, indicating no association with the executive inhibitory mechanisms involved in response suppression. Our findings also expand the limited body of literature on the mechanisms underlying responsiveness to and consumption of healthy foods. Moreover, while most studies on healthy eating have primarily focused on fruits and vegetables [see (86)], our results reveal distinct effects for fruit/vegetables and fish/lean meat, underscoring the need to take into account other healthy, nutrient-dense, food groups.

## Data Availability

The datasets presented in this study can be found in online repositories. The names of the repository/repositories and accession number(s) can be found at: Open Science Framework repository at https://osf.io/bjyx9/.
