# Supplementary material for: Nutritional habits, inhibitory control, and emotional reactivity to healthy and unhealthy food cues in non-obese female students: insights from heart rate variability
Source: Front Nutr. 2025 Sep 3;12:1622087. doi: 10.3389/fnut.2025.1622087 (PMC12442432; doi:10.3389/fnut.2025.1622087)
Supplement: Supplementary file 2 [file Table_2.docx]

**Table S2.** Summary of the hierarchical regression analysis for variables predicting emotional reactivity to fish/lean meat food.

| **Model** | **Predictors** | **Beta** | **t** | **p** | **R^2^** | **∆R^2^** |
| --- | --- | --- | --- | --- | --- | --- |
| **Step 1*** | BMI | 0.244 | 1.637 | 0.110 | 0.192 |  |
|  | Food deprivation | -0.367 | -2.480 | 0.018 |  |  |
|  | Hunger | -0.188 | -1.271 | 0.211 |  |  |
| **Step 2** | BMI | 0.227 | 1.451 | 0.155 | 0.196 | 0.004 |
|  | Food deprivation | -0.364 | -2.434 | 0.020 |  |  |
|  | Hunger | -0.175 | -1.154 | 0.256 |  |  |
|  | HRV | 0.068 | 0.439 | 0.663 |  |  |

*Note:* * significant model(s). BMI = body mass index; HRV = heart rate variability.
