# Supplementary material for: Nutritional habits, inhibitory control, and emotional reactivity to healthy and unhealthy food cues in non-obese female students: insights from heart rate variability
Source: Front Nutr. 2025 Sep 3;12:1622087. doi: 10.3389/fnut.2025.1622087 (PMC12442432; doi:10.3389/fnut.2025.1622087)
Supplement: Supplementary file 3 [file Table_3.docx]

**Table S3.** Summary of the hierarchical regression analysis for variables predicting emotional reactivity to savory junk food.

| **Model** | **Predictors** | **Beta** | **t** | **p** | **R^2^** | **∆R^2^** |
| --- | --- | --- | --- | --- | --- | --- |
| **Step 1*** | BMI | -0.166 | -1.107 | 0.275 | 0.185 |  |
|  | Food deprivation | -0.240 | -1.614 | 0.115 |  |  |
|  | Hunger | 0.331 | 2.233 | 0.032 |  |  |
| **Step 2** | BMI | -0.159 | -1.011 | 0.319 | 0.186 | 0.001 |
|  | Food deprivation | -0.241 | -1.598 | 0.118 |  |  |
|  | Hunger | 0.326 | 2.134 | 0.040 |  |  |
|  | HRV | -0.027 | -0.174 | 0.863 |  |  |

*Note:* * significant model(s). BMI = body mass index; HRV = heart rate variability.
