# Supplementary material for: Nutritional habits, inhibitory control, and emotional reactivity to healthy and unhealthy food cues in non-obese female students: insights from heart rate variability
Source: Front Nutr. 2025 Sep 3;12:1622087. doi: 10.3389/fnut.2025.1622087 (PMC12442432; doi:10.3389/fnut.2025.1622087)
Supplement: Supplementary file 4 [file Table_4.docx]

**Table S4.** Summary of the hierarchical regression analysis for variables predicting Go reaction times for fish/lean meat food.

| **Model** | **Predictors** | **Beta** | **t** | **p** | **R^2^** | **∆R^2^** |
| --- | --- | --- | --- | --- | --- | --- |
| **Step 1** | BMI | -0.156 | -0.988 | 0.330 | 0.095 |  |
|  | Food deprivation | 0.283 | 1.810 | 0.078 |  |  |
|  | Hunger | -0.060 | -0.385 | 0.702 |  |  |
| **Step 2** | BMI | -0.197 | -1.202 | 0.237 | 0.117 | 0.022 |
|  | Food deprivation | 0.344 | 2.040 | 0.049 |  |  |
|  | Hunger | -0.029 | -0.181 | 0.857 |  |  |
|  | Emotional reactivity to fish/lean meat | 0.166 | 0.969 | 0.339 |  |  |
| **Step 3*** | BMI | -0.302 | -1.973 | 0.056 | 0.289 | 0.172 |
|  | Food deprivation | 0.348 | 2.267 | 0.029 |  |  |
|  | Hunger | 0.044 | 0.301 | 0.765 |  |  |
|  | Emotional reactivity to fish/lean meat | 0.133 | 0.850 | 0.401 |  |  |
|  | HRV | 0.434 | 2.948 | 0.006 |  |  |

*Note:* * significant model(s). BMI = body mass index; HRV = heart rate variability.
