# Supplementary material for: Nutritional habits, inhibitory control, and emotional reactivity to healthy and unhealthy food cues in non-obese female students: insights from heart rate variability
Source: Front Nutr. 2025 Sep 3;12:1622087. doi: 10.3389/fnut.2025.1622087 (PMC12442432; doi:10.3389/fnut.2025.1622087)
Supplement: Supplementary file 5 [file Table_5.docx]

**Table S5.** Summary of the hierarchical regression analysis for variables predicting reaction times to Go stimuli for savory junk food.

| **Model** | **Predictors** | **Beta** | **t** | **p** | **R^2^** | **∆R^2^** |
| --- | --- | --- | --- | --- | --- | --- |
| **Step 1** | BMI | -0.056 | -0.351 | 0.728 | 0.078 |  |
|  | Food deprivation | 0.145 | 0.920 | 0.363 |  |  |
|  | Hunger | -0.234 | -1.487 | 0.145 |  |  |
| **Step 2** | BMI | -0.006 | -0.037 | 0.970 | 0.152 | 0.074 |
|  | Food deprivation | 0.218 | 1.371 | 0.179 |  |  |
|  | Hunger | -0.334 | -2.051 | 0.047 |  |  |
|  | Emotional reactivity to savory junk food | 0.301 | 1.798 | 0.080 |  |  |
| **Step 3*** | BMI | -0.102 | -0.671 | 0.506 | 0.280 | 0.128 |
|  | Food deprivation | 0.234 | 1.579 | 0.123 |  |  |
|  | Hunger | -0.269 | -1.746 | 0.089 |  |  |
|  | Emotional reactivity to savory junk food | 0.313 | 1.997 | 0.053 |  |  |
|  | HRV | 0.375 | 2.534 | 0.016 |  |  |

*Note:* * significant model(s). BMI = body mass index; HRV = heart rate variability.
