# Supplementary material for: Nutritional habits, inhibitory control, and emotional reactivity to healthy and unhealthy food cues in non-obese female students: insights from heart rate variability
Source: Front Nutr. 2025 Sep 3;12:1622087. doi: 10.3389/fnut.2025.1622087 (PMC12442432; doi:10.3389/fnut.2025.1622087)
Supplement: Supplementary file 6 [file Table_6.docx]

**Table S6.** Summary of the hierarchical regression analysis for variables predicting reaction times to Go stimuli for sweet junk food.

| **Model** | **Predictors** | **Beta** | **t** | **p** | **R^2^** | **∆R^2^** |
| --- | --- | --- | --- | --- | --- | --- |
| **Step 1** | BMI | -0.114 | -0.739 | 0.465 | 0.138 |  |
|  | Food deprivation | 0.329 | 2.151 | 0.038 |  |  |
|  | Hunger | -0.168 | -1.103 | 0.277 |  |  |
| **Step 2** | BMI | -0.116 | -0.749 | 0.459 | 0.158 | 0.020 |
|  | Food deprivation | 0.276 | 1.688 | 0.100 |  |  |
|  | Hunger | -0.159 | -1.038 | 0.306 |  |  |
|  | Emotional reactivity to sweet junk food | 0.15 | 0.931 | 0.358 |  |  |
| **Step 3*** | BMI | -0.212 | -1.415 | 0.166 | 0.281 | 0.124 |
|  | Food deprivation | 0.296 | 1.932 | 0.061 |  |  |
|  | Hunger | -0.093 | -0.635 | 0.529 |  |  |
|  | Emotional reactivity to sweet junk food | 0.132 | 0.871 | 0.389 |  |  |
|  | HRV | 0.369 | 2.491 | 0.017 |  |  |

*Note:* * significant model(s). BMI = body mass index; HRV = heart rate variability.
