# Supplementary material for: Nutritional habits, inhibitory control, and emotional reactivity to healthy and unhealthy food cues in non-obese female students: insights from heart rate variability
Source: Front Nutr. 2025 Sep 3;12:1622087. doi: 10.3389/fnut.2025.1622087 (PMC12442432; doi:10.3389/fnut.2025.1622087)
Supplement: Supplementary file 7 [file Table_7.docx]

**Table S7.** Summary of the hierarchical regression analysis for variables predicting the habitual consumption of fruit/vegetables.

| **Model** | **Predictors** | **Beta** | **t** | **p** | **R^2^** | **∆R^2^** |
| --- | --- | --- | --- | --- | --- | --- |
| **Step 1*** | BMI | 0.270 | 1.852 | 0.072 | 0.364 |  |
|  | Physical activity | 0.424 | 2.905 | 0.006 |  |  |
| **Step 2*** | BMI | 0.245 | 1.620 | 0.114 | 0.389 | 0.026 |
|  | Physical activity | 0.463 | 3.087 | 0.004 |  |  |
|  | Emotional reactivity to fruit/vegetables | 0.152 | 1.149 | 0.258 |  |  |
|  | Inhibitory control over unhealthy food | -0.052 | -0.396 | 0.695 |  |  |
| **Step 3*** | BMI | 0.196 | 1.346 | 0.187 | 0.460 | 0.070 |
|  | Physical activity | 0.433 | 3.007 | 0.005 |  |  |
|  | Emotional reactivity to fruit/vegetables | 0.076 | 0.576 | 0.568 |  |  |
|  | Inhibitory control over unhealthy food | -0.018 | -0.142 | 0.888 |  |  |
|  | HRV | 0.286 | 2.162 | 0.037 |  |  |

*Note:* * significant model(s). BMI = body mass index; HRV = heart rate variability.
