# Supplementary material for: Nutritional habits, inhibitory control, and emotional reactivity to healthy and unhealthy food cues in non-obese female students: insights from heart rate variability
Source: Front Nutr. 2025 Sep 3;12:1622087. doi: 10.3389/fnut.2025.1622087 (PMC12442432; doi:10.3389/fnut.2025.1622087)
Supplement: Supplementary file 8 [file Table_8.docx]

**Table S8.** Summary of the hierarchical regression analysis for variables predicting the habitual consumption of fish/lean meat.

| **Model** | **Predictors** | **Beta** | **t** | **p** | **R^2^** | **∆R^2^** |
| --- | --- | --- | --- | --- | --- | --- |
| **Step 1*** | BMI | 0.188 | 1.188 | 0.242 | 0.247 |  |
|  | Physical activity | 0.378 | 2.383 | 0.022 |  |  |
| **Step 2*** | BMI | 0.135 | 0.920 | 0.364 | 0.410 | 0.163 |
|  | Physical activity | 0.366 | 2.532 | 0.016 |  |  |
|  | Emotional reactivity to fish/lean meat | 0.408 | 3.194 | 0.003 |  |  |
|  | Inhibitory control over unhealthy food | -0.023 | -0.182 | 0.856 |  |  |
| **Step 3*** | BMI | 0.118 | 0.787 | 0.437 | 0.416 | 0.006 |
|  | Physical activity | 0.363 | 2.485 | 0.018 |  |  |
|  | Emotional reactivity to fish/lean meat | 0.400 | 3.077 | 0.004 |  |  |
|  | Inhibitory control over unhealthy food | -0.013 | -0.096 | 0.924 |  |  |
|  | HRV | 0.075 | 0.565 | 0.575 |  |  |

*Note:* * significant model(s). BMI = body mass index; HRV = heart rate variability.
