# Supplementary material for: Nutritional habits, inhibitory control, and emotional reactivity to healthy and unhealthy food cues in non-obese female students: insights from heart rate variability
Source: Front Nutr. 2025 Sep 3;12:1622087. doi: 10.3389/fnut.2025.1622087 (PMC12442432; doi:10.3389/fnut.2025.1622087)
Supplement: Supplementary file 9 [file Table_9.docx]

**Table S9.** Summary of the hierarchical regression analysis for variables predicting the habitual consumption of sweet junk food.

| **Model** | **Predictors** | **Beta** | **t** | **p** | **R^2^** | **∆R^2^** |
| --- | --- | --- | --- | --- | --- | --- |
| **Step 1*** | BMI | 0.232 | 1.479 | 0.147 | 0.264 |  |
|  | Physical activity | -0.584 | -3.725 | 0.001 |  |  |
| **Step 2*** | BMI | 0.171 | 1.090 | 0.283 | 0.334 | 0.070 |
|  | Physical activity | -0.562 | -3.377 | 0.002 |  |  |
|  | Emotional reactivity to sweet junk food | 0.199 | 1.401 | 0.170 |  |  |
|  | Inhibitory control over sweet junk food | 0.185 | 1.306 | 0.200 |  |  |
| **Step 3*** | BMI | 0.226 | 1.479 | 0.148 | 0.405 | 0.071 |
|  | Physical activity | -0.542 | -3.394 | 0.002 |  |  |
|  | Emotional reactivity to sweet junk food | 0.215 | 1.579 | 0.123 |  |  |
|  | Inhibitory control over sweet junk food | 0.170 | 1.252 | 0.219 |  |  |
|  | HRV | -0.274 | -2.065 | 0.046 |  |  |

*Note:* * significant model(s). BMI = body mass index; HRV = heart rate variability.
